# Coumarins from *Edgeworthia chrysantha*

**DOI:** 10.3390/molecules19022042

**Published:** 2014-02-13

**Authors:** Xing-Nuo Li, Sheng-Qiang Tong, Dong-Ping Cheng, Qing-Yong Li, Ji-Zhong Yan

**Affiliations:** College of Pharmaceutical Science, Zhejiang University of Technology, 18 Chaowang Road, Hangzhou 310014, China

**Keywords:** coumarin, *Edgeworthia chrysantha*, flower buds, edgeworic acid

## Abstract

A new coumarin, edgeworic acid (**1**), was isolated from the flower buds of *Edgeworthia chrysantha*, together with the five known coumarins umbelliferone (**2**), 5,7-dimethoxycoumarin (**3**), daphnoretin (**4**), edgeworoside C (**5**), and edgeworoside A (**6**). Their structures were established on the basis of spectral data, particularly by the use of 1D NMR and several 2D shift-correlated NMR pulse sequences (^1^H-^1^H COSY, HSQC and HMBC), in combination with acetylation reactions.

## 1. Introduction

The genus *Edgeworthia* (Thymelaeaceae) consists of five species distributed around the World, which are native to China, India, Japan, and southeast of America [1]. *E. chrysantha* is widely distributed and is endemic to South and East China [1]. The bark of *E. chrysantha* is used as “Zushima” in some local areas in China for the treatment of traumatic injury and rheumatism [2,3], and the fiber of the stem bark is a raw materials for making high quality paper [1]. The flower buds are often used as the traditional Chinese medicine “Mimenghua” for the treatment of ophthalmalgia and delacrimation [1,4].

**Figure 1:**
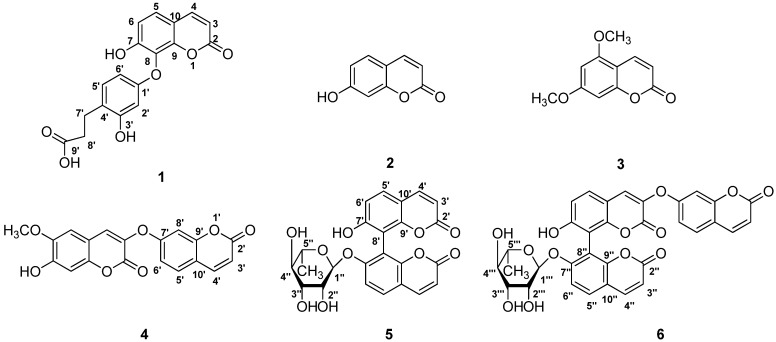
Chemical structures of compounds **1**–**6**.

Phytochemical studies have revealed that *E. chrysantha* contains various constituents, such as coumarins [5,6,7,8], flavonoids [9,10,11], terpenes [12,13,14] and lignans [15,16]. Among them coumarins are generally considered as the major anti-inflammatory and analgesia bioactive constituents [17]. In our continuing search for pharmacological and structurally interesting substances from the flower buds of *E. chrysantha*, a new coumarin, edgeworic acid (**1**), has been isolated, along with the five known coumarins umbelliferone (**2**) [18], 5,7-dimethoxycoumarin (**3**) [19] daphnoretin (**4**) [20], edgeworoside C (**5**) [18], and edgeworoside A (**6**) [18] (Figure 1). The structure of the new compound was elucidated by spectroscopic methods and confirmed by acetylation.

## 2. Results and Discussion

Compound **1** was obtained as a white powder. The molecular formula C_18_H_14_O_7_ was determined by HR-ESI-MS ([M−H]^−^ peak at *m/z* 341.0752), indicating 12 degrees of unsaturation. The ^13^C-NMR and DEPT spectra resolved 18 carbon signals, which were classified by chemical shifts and HSQC spectrum as two carbonyl groups [*δ*_C_ 174.6 (C-9'), *δ*_C_ 160.2 (C-2)], seven *sp*^2^ quaternary carbons [*δ*_ C_ 157.2 (C-1'), *δ*_C_ 156.3 (C-3'), *δ*_C_ 154.8 (C-7), *δ*_C_ 148.8 (C-9), *δ*_C_ 128.6 (C-8), *δ*_C_ 121.0 (C-4'), *δ*_C_ 112.5 (C-10)], seven *sp*^2^ methines [*δ*_C_ 145.1 (C-4), *δ*_C_ 130.5 (C-5'), *δ*_C_ 125.7 (C-5), *δ*_C_ 114.1 (C-6), *δ*_C_ 112.1 (C-3), *δ*_C_ 105.7 (C-6'), *δ*_C_ 102.0 (C-2')], two *sp*^3^ methylenes [*δ*_C_ 34.3 (C-8'), *δ*_C_ 25.3 (C-7')] (Table 1).

The IR spectrum exhibited vibration bands for free hydroxyl (3334 cm^−1^), carboxyl (3207, 1732 cm^−1^), conjugated carbonyl (1692 cm^−1^), and aromatic (1610, 1519, 1448 cm^−1^) functionalities. The UV spectrum exhibited a maximum absorption at 322 nm. According to the data mentioned above, it is suggested that compound **1** has a coumarin skeleton. This was further supported by the ^1^H-NMR signals [*δ*_H_ 8.01 (1H, d, *J* = 9.2 Hz, H-4); *δ*_H_ 6.25 (1H, d, *J* = 9.2 Hz, H-3)] (Table 1), and ^13^C-NMR signals [*δ*_C_ 160.2 (C-2); *δ*_C_ 145.1 (C-4); *δ*_C_ 112.1 (C-3)] [8].

**Figure 2:**
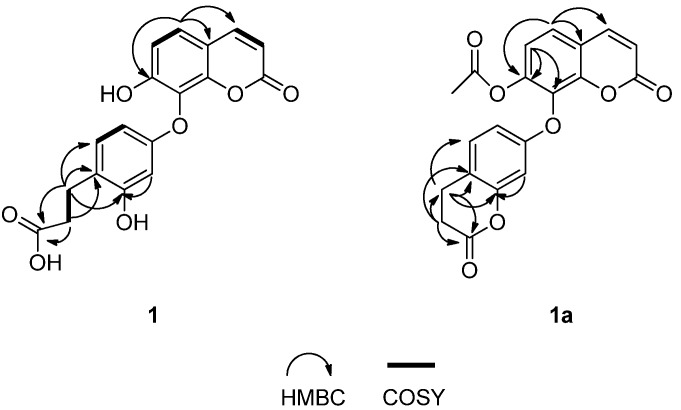
Key HMBC and ^1^H-^1^H COSY correlations of **1** and **1a**.

The ^1^H-NMR spectrum of **1** (Table 1) showed the presence of a set of *ortho*-coupled aromatic signals [*δ*_H_ 7.46 (1H, d, *J* = 8.4 Hz, H-5); *δ*_H_ 6.99 (1H, d, *J* = 8.4 Hz, H-6)]. The ^1^H-NMR data also showed an ABX-type coupling system [*δ*_H_ 6.97 (1H, d, *J* = 8.4 Hz, H-5'); *δ*_H_ 6.29 (1H, d, *J* = 2.4 Hz, H-2'); *δ*_H_ 6.24 (1H, overlapped, H-6')]. The signals at *δ*_H_ 2.68 (2H, t, *J* = 7.2 Hz, H-7'), *δ*_C_ 25.3 (C-7'), *δ*_H_ 2.43 (2H, t, *J* = 7.2 Hz, H-8'), *δ*_C_ 34.3 (C-8') showed the existence of a 3-propionic acid group [8], which was confirmed by the HSQC, HMBC, and ^1^H-^1^H COSY spectra. (Figure 2) In the HMBC spectrum, the ^1^H-NMR signal at *δ*_H_ 2.43 (H-8') was correlated to ^13^C-NMR signal at *δ*_C_ 121.0 (C-4'), and the ^1^H-NMR signal at *δ*_H_ 2.68 (H-7') showed correlations with ^13^C-NMR signals at *δ*_C_ 156.3 (C-3'), 121.0 (C-4') and 130.5 (C-5'), indicating that 3-propionic acid group was located at the C-4' position. (Figure 2) The aromatic H-atom at *δ*_H_ 7.46, which correlated with 154.8 (C-7), 148.8 (C-9), 145.1 (C-4) in the HMBC spectrum (Figure 2), could be assigned to H-5. Since it coupled with the H-6, the substitution site at the coumarin skeleton was established at C-7 and C-8.

**Table 1:** ^1^H- and ^13^C-NMR data of **1** and **1a** (*δ* in ppm and *J* in Hz).

No.	1		No.	1a	
	*δ*_H_	*δ*_C_		*δ*_H_	*δ*_C_
2	—	160.2 s	2	—	159.3 s
3	6.25 (d, 9.2)	112.1 d	3	6.53 (d, 9.6)	116.5 d
4	8.01 (d, 9.2)	145.1 d	4	8.13 (d, 9.6)	144.4 d
5	7.46 (d, 8.4)	125.7 d	5	7.71 (d, 8.4)	125.6 d
6	6.99 (d, 8.4)	114.1 d	6	7.33 (d, 8.4)	120.3 d
7	—	154.8 s	7	—	146.1 s
8	—	128.6 s	8	—	133.3 s
9	—	148.8 s	9	—	147.7 s
10	—	112.5 s	10	—	118.9 s
1'	—	157.2 s	1'	—	157.0 s
2'	6.29 (d, 2.4)	102.0 d	2'	6.65 (d, 2.4)	104.0 d
3'	—	156.3 s	3'	—	152.7 s
4'	—	121.0 s	4'	—	118.1 s
5'	6.97 (d, 8.4)	130.5 d	5'	7.24 (d, 8.4)	129.6 d
6'	6.24 (overlapped)	105.7 d	6'	6.69 (dd, 2.4, 8.4)	111.2 d
7'	2.68 (t, 7.2)	25.3 t	7'	2.94 (t, 7.2)	22.5 t
8'	2.43 (t, 7.2)	34.3 t	8'	2.78 (t, 7.2)	28.9 t
9'	—	174.6 s	9'	—	168.4 s
			Ac	2.15 (s)	20.6 q 168.5 s

According to the molecular formula of **1**, there were only two phenolic hydroxyl groups left. Unfortunately, no phenolic hydroxyl groups signals appeared in the ^1^H-NMR spectrum, so the locations of the phenolic hydroxyl groups were confirmed by acetylation. The acetylated derivative of **1** (compound **1a**) was obtained as a white powder. The molecular formula C_20_H_16_O_7_ was determined by ESI-MS ([M+H]^+^ peak at *m/z* 367.0), indicating 13 degrees of unsaturation. By comparing the MS data of **1a** with those of **1**, it was presumed that a six membered lactone ring was formed during the acetylation reaction. The comparison of the ^13^C-NMR data of **1** with those of **1a** (Table 1) revealed that the signals of C-6 and C-8 were shifted downfield in the range of *δ* 5–6 ppm, the signal of C-7 was shifted upfield by *δ* 8.7 ppm, and the signal of C-3' was shifted upfield by *δ* 3.6 ppm. According to the analysis mentioned above, one of the phenolic hydroxyl groups was deduced to be at C-7 [21], and another one was located at C-3', which was confirmed by the HMBC spectrum. (Figure 2).

The five known coumarins were identified as umbelliferone (**2**) [18], 5,7-dimethoxycoumarin (**3**) [19] daphnoretin (**4**) [20], edgeworoside C (**5**) [18], and edgeworoside A (**6**) [18], by interpretation of their spectroscopic data and comparison with literature values.

## 3. Experimental

### 3.1. General

All chemical solvents used were of analytical grade. Column chromatography (CC): MCI gel (Mitsubishi Chemical Co., Tokyo, Japan); Sephadex LH-20 (Pharmacia Fine Chemical Co. Ltd., Uppsala, Sweden); silica gel (Qingdao Marine Chemical Group Co., Qingdao, China; 200–300 and 400–600 mesh). HPLC: Agilent 1100 series (Agilent Technologies, Palo Alto, CA, USA) equipped with an Agilent DAD spectrophotometer and an Alltima-C18 reversed-phase column (5 µm, 250 × 10 mm) with an Eclipse XDB-C18 guard column. IR spectra: Nicolet-Magna-FT-IR 750 spectrometer (Thermo Scientific, Waltham, MA, USA). UV spectra: Shimadzu UV-2450 spectrophotometer (Shimadzu, Kyoto, Japan). LR- and HR-ESI-MS: Finnigan LCQ-Deca (Thermo Scientific, Waltham, MA, USA) and Waters Micromass Q-TOF-Ultima mass spectrometers (Waters, Milford, MA, USA). ^1^H- and ^13^C-NMR spectra were recorded on a Bruker Avance-400 spectrometer (Bruker, Karlsruhe, Germany) (^1^H- at 400 MHz, ^13^C- at 100 MHz) in DMSO-*d_6_* at room temperature (22 °C). Chemical shifts are reported in ppm (*δ*), relative to tetramethylsilane as internal standard, and coupling constants are in Hertz.

### 3.2. Plant Material

The flower buds of *E. chrysantha* were collected in a garden of Lishui, Zhejiang Province, China, in February 2008. The plants were authenticated by Dr. Chu Chu, Zhejiang University of Technology, China. A voucher specimen (TCM 2008-026) was deposited in College of Pharmaceutical Science, Zhejiang University of Technology.

### 3.3. Extraction and Isolation

The air-dried material (10 kg) were extracted at room temperature and for 36 h × 3 with 95% (*v/v*) EtOH (5 L × 3) to give, after removal of the solvent, 220 g of crude extract which was dissolved in 4 L of H_2_O to form a suspension and successively partitioned with petroleum ether (60–90 °C) (3,000 mL × 3), ethyl acetate (3,000 mL × 3) and *n*-butanol (3,000 mL × 3). The ethyl acetate extracts (14 g) were chromatographed on a silica gel column (petroleum ether/ethyl acetate, 4:1–0:1 *v/v*) to give eight fractions 1–8. Fraction 1 (4:1 *v/v*; 1.2 g) was separated on a silica gel H column (petroleum ether/ethyl acetate, 6:1 *v/v*) to afford three fractions 1'–3'. Subfraction 3' (100 mg) was chromatographed on a silica gel H column (petroleum ether/acetone, 6:1 *v/v*) to afford compound **2** (28 mg) and **3** (27 mg). Fraction 3 (3:1 *v/v*; 0.2 g) was recrystallized with methanol to give compound **4** (45 mg). Fraction 4 (2.5:1 *v/v*; 1.0 g) was separated on a silica gel H column (CHCl_3_/acetone, 6:1 *v/v*) to afford compound **1** (43 mg). Fraction 7 (1:1 *v/v*; 3.8 g) was chromatographed on a MCI gel column (MeOH/H_2_O, 1:9–8:2 *v/v*) to give three fractions 1'–3'. Subfraction 1' (100 mg) was recrystallized from methanol to afford compound **5** (55 mg). Subfraction 3' (80 mg) was subjected to Sephadex LH-20 column chromatography (3 × 100 cm) eluted with CHCl_3_/MeOH (1:1 *v/v*) to remove the pigments and finally purified by semipreparative-HPLC using MeOH/H_2_O (64:36 *v/v*, 25 °C, 3.0 ml/min) to afford compound **6** (13 mg, t_R_ = 17.38 min).

### 3.4. Acetylation of Edgeworic acid (**1**)

A mixture of compound **1** (20 mg), Ac_2_O (5 mL), and pyridine (5 mL) was stirred at room temperature overnight. The resulting solution was concentrated under vacuum. The residue was dissolved in ethyl acetate and washed with water (5 mL). The product was purified on a silica gel column (*n*-hexane/ethyl acetate, 3:2 *v/v*) to afford **1a** (15 mg).

*Acetylated derivative of edgeworic acid* (**1a**). White powder; ESI-MS (+) *m/z* 367.0 [M+H]^+^; ^1^H-NMR (DMSO-*d_6_*) and ^1^^3^C-NMR (DMSO-*d_6_*) data: see Table 1.

### 3.5. Spectral Data

*Edgeworic acid* (**1**). White powder; UV (MeOH) λ_max_ (log ε): 322 (4.20); IR (KBr) *v*_max_: 3,334, 3,207, 1732, 1692, 1610, 1519, 1448 cm^−1^; HR-ESI-MS (−) *m/z* 341.0752 [M−H]^−^ (calcd. for C_18_H_13_O_7_, 341.0661); ^1^H-NMR (DMSO-*d_6_*) and ^1^^3^C-NMR (DMSO-*d_6_*) data: see Table 1.

*Umbelliferone* (**2**). Colorless needles; m.p.: 225–228 °C; ^1^H-NMR (DMSO-*d_6_*) *δ*: 10.6 (1H, s, 7-OH), 7.92 (1H, d, *J* = 9.5 Hz, H-4), 7.52 (1H, d, *J* = 8.5 Hz, H-5), 6.78 (1H, dd, *J* = 2.4, 8.5 Hz, H-6), 6.71 (1H, d, *J* = 2.4 Hz, H-8), 6.19 (1H, d, *J* = 9.5 Hz, H-3).

*5,7-Dimethoxycoumarin* (**3**). Colorless needles; m.p.: 144–145 °C; ^1^H-NMR (DMSO-*d_6_*) *δ*: 7.96 (1H, d, *J* = 9.6 Hz, H-4), 6.41 (1H, s, H-8), 6.28 (1H, s, H-6), 6.15 (1H, d, *J* = 9.6 Hz, H-3), 3.89 (3H, s, OCH_3_), 3.86 (3H, s, OCH_3_).

*Daphnoretin* (**4**). Yellow needles; m.p.: 223–225 °C; ^1^H-NMR (DMSO-*d_6_*) *δ*: 10.3 (1H, s, 7-OH), 8.05 (1H, d, *J* = 9.5 Hz, H-4'), 7.88 (1H, s, H-4), 7.72 (1H, d, *J* = 8.6 Hz, H-5'), 7.22 (1H, s, H-5), 7.20 (1H, d, *J* = 2.4 Hz, H-8'), 7.12 (1H, dd, *J* = 8.6, 2.4 Hz, H-6'), 6.87 (1H, s, H-8), 6.39 (1H, d, *J* = 9.5 Hz, H-3'), 3.82 (3H, s, 6-OCH_3_); ^1^^3^C-NMR (DMSO-*d_6_*) *δ*: 160.4 (C-2), 160.1 (C-2'), 157.4 (C-7'), 155.5 (C-9'), 150.8 (C-7), 147.9 (C-9), 146.1 (C-6), 144.5 (C-4'), 136.2 (C-3), 131.2 (C-4), 130.3 (C-5'), 114.9 (C-10'), 114.3 (C-3'), 113.9 (C-6'), 110.6 (C-10), 110.0 (C-5), 104.5 (C-8'), 103.2 (C-8), 56.5 (7-OCH_3_).

*Edgeworoside C* (**5**). White powder; ^1^H-NMR (DMSO-*d_6_*) *δ*: 10.6 (1H, br s, 7'-OH), 8.09 (1H, d, *J*= 9.2 Hz, H-4'), 8.03 (1H, d, *J* = 9.4 Hz, H-4), 7.78 (1H, d, *J* = 8.5 Hz, H-5'), 7.64 (1H, d, *J* = 8.3 Hz, H-5), 7.32 (1H, d, *J* = 8.5 Hz, H-6'), 7.00 (1H, d, *J* = 8.3 Hz, H-6), 6.33 (1H, d, *J* = 9.2 Hz, H-3'), 6.21 (1H, d, *J* = 9.4 Hz, H-3), 5.48 (1H, s, H-1''), 3.44 (1H, s, H-2''), 3.18 (2H, m, H-4'', 5''), 3.01 (1H, br s, H-3''), 1.06 (3H, d, *J* = 6.0 Hz, 5''-CH_3_); ^1^^3^C NMR (DMSO-*d_6_*) *δ*: 160.7 (C-2), 160.5 (C-2'), 159.7 (C-7), 157.5 (C-7'), 153.6 (C-9), 153.1 (C-9'), 145.4 (C-4'), 145.1 (C-4), 129.8 (C-5, 5'), 113.9 (C-10'), 113.4 (C-3'), 113.1 (C-6), 111.8 (C-6'), 111.6 (C-10), 111.5 (C-3), 110.4 (C-8'), 106.9 (C-8), 99.0 (C-1''), 71.9 (C-4''), 70.6 (C-3''), 70.4 (C-2''), 70.1 (C-5''), 18.3 (C-6'').

*Edgeworoside A* (**6**). White powder; ^1^H-NMR (DMSO-*d_6_*) *δ*: 10.5 (1H, br s, 7-OH), 8.10 (1H, d, *J* = 9.6 Hz, H-4''), 8.02 (1H, s, H-4), 8.01 (1H, d, *J* = 9.5 Hz, H-4'), 7.80 (1H, d, *J* = 8.9 Hz, H-5''), 7.69 (1H, d, *J* = 8.50 Hz, H-5′), 7.64 (1H, d, *J* = 8.6 Hz, H-5), 7.32 (1H, d, *J* = 8.9 Hz, H-6’’), 7.18 (1H, d, *J* = 2.2 Hz, H-8'), 7.07 (1H, dd, *J* = 8.5, 2.2 Hz, H-6'), 7.06 (1H, d, *J* = 8.6 Hz, H-6), 6.37 (1H, d, *J* = 9.5 Hz, H-3'), 6.34 (1H, d, *J* = 9.6 Hz, H-3''), 5.50 (1H, br s, H-1'''), 3.54 (1H, m, H-2'''), 3.29 (1H, m, H-5'''), 3.19 (1H, m, H-4'''), 2.94 (1H, m, H-3'''), 1.04 (3H, d, *J* = 6.2 Hz, 5'''-CH_3_).

## 4. Conclusions

A new coumarin, edgeworic acid (**1**), was isolated from the flower buds of *E. chrysantha* together with the five known compounds umbelliferone (**2**), 5,7-dimethoxycoumarin (**3**), daphnoretin (**4**), edgeworoside C (**5**), and edgeworoside A (**6**). Their structures were determined by spectroscopic analysis 1D-NMR, 2D-NMR and MS experiment combined with an acetylation reaction.
